# A workstation-integrated peer review quality assurance program: pilot study

**DOI:** 10.1186/1471-2342-13-19

**Published:** 2013-07-04

**Authors:** Margaret M O’Keeffe, Todd M Davis, Kerry Siminoski

**Affiliations:** 1 Department of Radiology and Diagnostic Imaging, University of Alberta, and Medical Imaging Consultants, 11010-101 Street, Edmonton, AB T5H 4B9, Canada; 2 Intelerad, Montreal, QC, Canada; 3 295 Midpark Way SE, Suite 380, Calgary, AB T2X 2A8, Canada

**Keywords:** Diagnostic errors, Diagnostic imaging, Peer review, Practice performance evaluation, Quality assurance, RADPEER

## Abstract

**Background:**

The surrogate indicator of radiological excellence that has become accepted is consistency of assessments between radiologists, and the technique that has become the standard for evaluating concordance is peer review. This study describes the results of a workstation-integrated peer review program in a busy outpatient radiology practice.

**Methods:**

Workstation-based peer review was performed using the software program Intelerad Peer Review. Cases for review were randomly chosen from those being actively reported. If an appropriate prior study was available, and if the reviewing radiologist and the original interpreting radiologist had not exceeded review targets, the case was scored using the modified RADPEER system.

**Results:**

There were 2,241 cases randomly assigned for peer review. Of selected cases, 1,705 (76%) were interpreted. Reviewing radiologists agreed with prior reports in 99.1% of assessments. Positive feedback (score 0) was given in three cases (0.2%) and concordance (scores of 0 to 2) was assigned in 99.4%, similar to reported rates of 97.0% to 99.8%. Clinically significant discrepancies (scores of 3 or 4) were identified in 10 cases (0.6%). Eighty-eight percent of reviewed radiologists found the reviews worthwhile, 79% found scores appropriate, and 65% felt feedback was appropriate. Two-thirds of radiologists found case rounds discussing significant discrepancies to be valuable.

**Conclusions:**

The workstation-based computerized peer review process used in this pilot project was seamlessly incorporated into the normal workday and met most criteria for an ideal peer review system. Clinically significant discrepancies were identified in 0.6% of cases, similar to published outcomes using the RADPEER system. Reviewed radiologists felt the process was worthwhile.

## Background

Even with the recognition that “quality in health care is a complex and rather vague concept” [1], the quality of reporting in diagnostic radiology has become an important issue for radiology professional associations and for regulatory bodies [2-9]. The definitive quality assessment approach in radiology is correlation of radiological findings with the ultimate clinical outcome [1,2,4,10]. Applying this approach across all of diagnostic radiology is cost-prohibitive because of the long timeframes often needed to ascertain the clinical diagnosis and because of the manpower that would be involved [1,4,8,11,12]. In some branches of medicine, including interventional radiology, objective clinical or laboratory parameters are used as markers of quality [1,2,8,10]. This methodology is not applicable to most areas in diagnostic radiology. The surrogate indicator of radiological excellence that has become accepted is consistency of assessments between radiologists, and the technique that has become the standard for evaluating concordance is peer review [1-4,7,8,13]. The underlying intent is that the results of such reviews should act as an impetus for self-learning and other educational activities that ultimately lead to improved radiological performance and better patient outcomes [2,4,7,9,13-16].

There are several peer review approaches in use. One example is 360-degree feedback where radiologists have questionnaires completed by fellow radiologists, referring physicians, other healthcare staff, and patients, assessing a variety of topics including the quality of radiological assessments [6]. A second type of peer review is double reading, in which a random selection of routine cases is chosen for a second read, with any discrepancies between reads adjudicated by a third radiologist [5,7,11]. By far the most-used peer review approach in North America is the RADPEER program of the American College of Radiology (ACR) [3,4]. In this scheme, members of participating radiology groups evaluate prior images and reports of cases currently being reported and rate the quality of the original interpretation [3,4]. A four-point rating scale was originally used, with recent modifications being the addition of score 0 to assign positive feedback, and the incorporation of the option to designate clinical significance [3,4]. The RADPEER evaluation is submitted to the ACR, which provides statistical evaluation of ratings for individual radiologists and for facilities. More than 10,000 radiologists have participated in this program, representing about one-third of radiologists in the United States [3,4,17].

The RADPEER program has several practical drawbacks that have limited participation by the majority of radiologists in the US and that make the implementation of similar national programs in other countries problematic [11]. The first limitation is that case selection is not random [11,18]. The RADPEER program assumes that all active cases with prior studies will be evaluated, but in reality this is generally not the case. When time pressures arise, prior cases are often not evaluated, and it is likely that it is the most complex cases that are not reviewed [4,11,13]. The second problem is that additional time commitments are required to complete either machine-readable cards or internet assessment forms for each evaluated case. Another issue is that participating radiologists and practices are dependent on a third party (the ACR) for statistical compilation and analysis, with an associated time delay [4,19]. Finally, the RADPEER program does not mandate discrepancy case rounds, recognized as a valuable addition to peer review programs [11,13,15,18,20,21].

The concepts involved in performance and evaluation of peer review are evolving, but there is consensus on the characteristics of good peer review [3,4,13,15,18]. We have instituted a pilot peer review program to assess the feasibility of such an approach in a large multi-facility radiology practice. The pilot project had the following key characteristics: (1) the review process was workstation-based and integrated into reporting software so that it was seamlessly incorporated into the normal workday; (2) the process was practice-integrated in that the images chosen for review were prior studies related to cases currently being reported, as in the RADPEER system, with the review performed during the reporting process; (3) the cases for review were randomly generated by the software to avoid selection bias; (4) assessment data was immediately locally available and could be used for discrepancy rounds and case-sharing; and (5) discrepancy rounds and case-sharing of discrepant cases were incorporated into the program. In this paper we describe the results of our pilot program and compare them to published results of studies that have also used the RADPEER scoring system.

## Methods

### Peer review software

This study was performed between September 2009 and March 2010 in community-based outpatient clinic settings. Ethics approval was not required by the Health Research Ethics Board of the University of Alberta as the data was collected as part of a quality control program. Workstation-based peer review was performed using the software Intelerad Peer Review (Intelerad, Montreal, Quebec), which was integrated into the Radiology Information System (RIS; Intelerad). The program used two main steps to determine whether a case underwent review. The first step was determining whether a prior study of a patient whose current images were being evaluated was appropriate for peer review. A case being actively reported was randomly chosen and a paradigm was followed to determine if a prior study was available, was in the same modality as the current study, had either the same fee code or was of the same body area, and had been performed more than four days before the current study for most tests or within nine months of the current study for an obstetrical study.

The adopted terminology used here refers to the physician reporting the current study who is flagged to perform a peer review of prior studies as the reviewing radiologist. The physician who reported the prior study that is undergoing assessment is referred to as the interpreting radiologist [4]. The second step in the computerized selection process involved assessing targets for both the reviewing radiologist and the interpreting radiologist. Reviewing radiologists were each assigned a daily target for the number of peer reviews to perform (four to ten cases per full outpatient clinical reporting day). If the reviewing radiologist had performed fewer reviews than the daily target, the case was designated for possible peer review. If the reviewing radiologist had already met the daily target, or if the prior imaging had been interpreted by the reviewing radiologist, the case was not assessed. Interpreting radiologists were assigned a monthly maximum of 100 peer reviews per modality. If the number of peer reviewed cases for an interpreting radiologist was less than this for the modality of the case under consideration, the case was assigned to the reviewing radiologist for review. If the interpreting radiologist’s daily target in that modality had already been met, the case was discarded and not reviewed.

When a case met these criteria, the reviewing radiologist was notified that a relevant prior study had been chosen for peer review. The reviewing radiologist then had the option of reviewing the prior study immediately, reviewing it after dictating the current case, or reviewing it at a later date. To review the case, previous images were viewed in the picture archiving and communication system (PACS: Agfa Impax 6.3.1, Agfa Healthcare Corporation, Greenville, SC, United States) and the prior report was assessed and given a score.

### Quality assessment scoring

The reviewing radiologist performing peer review of an identified prior study evaluated the current and prior images and the prior report, then assigned a quality score to the prior report using the modified ACR RADPEER system [3-5,11,19]. A score of 0 indicates positive feedback. A score of 1 was assigned when the reviewer agreed with the original report. Scores 2, 3, and 4 indicated increasing disagreement with the prior report: 2 = error in diagnosis–not usually made; 3 = error in diagnosis – should usually be made; 4 = error in diagnosis – should almost always be made [3,4]. Cases were drawn from the modalities of general radiography, fluoroscopy, mammography, nuclear medicine, and ultrasonography, which encompass 89.1% of the clinic-based caseload of the practice. CT and MRI were not included in this pilot study as in our health region these are primarily hospital-based procedures rather than community-based. For comparison to published data, case scores were grouped as follows based on terminology in the literature: non-discrepant (scores in the range of 0 and 1), concordant (scores of 0 to 2), discrepant (2 to 4), and clinically significant discrepancy (3 and 4) [4,19,22].

### Radiologist characteristics and clinical setting

A total of 10 radiologists participated as peer reviewers. The mean amount of time since accreditation in general radiology was 17.5 years (SD, 7.4 years) with a range from 2 to 25 years. Subspecialty accreditation in the imaging modalities relevant to this pilot study included ultrasonography (10/10), mammography (6/10), cardiac echocardiography (6/10), and nuclear medicine (1/10). In subspecialty cases, both the interpreting and reviewing radiologists were accredited in that subspecialty.

Medical Imaging Consultants (MIC) of Edmonton, Alberta is a partnership of approximately 80 general and subspecialty diagnostic radiologists. The mean amount of time since accreditation in general radiology was 15.6 years (SD, 8.9 years) with a range from 1 to 37 years. Subspecialty accreditation in the imaging modalities relevant to this pilot study included ultrasonography (100%), mammography (18.8%), cardiac echocardiography (15%), and nuclear medicine (12%). Within these subspecialty modalities, reporting was done only by those physicians accredited in the particular modality. Twenty-three radiologists in the practice spent less than one day per month in clinic work, so that 57 radiologists served as the principle interpreting radiologists in this pilot study. A survey was completed by these radiologists at the end of the pilot period.

### Approach to discrepant cases

All interpreting radiologists were informed of the results of each of their peer reviewed cases. When a case was scored 2, 3, or 4, the interpreting radiologist was required to reassess the original images and report. The interpreting radiologist also had the option of seeking a subsequent review by another radiologist, but this was not requested for any case during the pilot study. Aggregate peer review statistics were available to all radiologists. A quality assurance (QA) committee reviewed all score 3 and 4 cases. Selected score 3 and 4 cases were presented at discrepancy rounds and made available to the membership as virtual discrepancy rounds on a secure website [11,21].

### Statistics

Values are expressed as raw numbers and percentages with 95% confidence intervals (CI). Data was processed using SPSS version 12.0 (SPSS).

## Results

A total of 2,241 cases were chosen for review (Table 1). The median number per reviewing physician was 216 cases, with a range from 39 to 529. Reviews were performed on 1,705 of these cases (76%; 95% CI, 74 to 78%). The median proportion of completed reviews per radiologist was 82% with a range from 26% to 100%. The mean non-completion rate was 24% (95% CI, 22 to 26%), while the median non-completion rate was 18%, with a range from 0% to 74%. Four radiologists completed less than two-thirds of assigned cases. Of the reviewed cases, 94% (95% CI, 93 to 95%) were completed within 15 minutes of assignment. Fluoroscopy made up the lowest fraction of the total assigned cases at 3% and general radiography the highest at 34% (Table 2); these comprised approximately similar proportions of the total tests in each modality done by MIC in this time period.

**Table 1:** Assigned and reviewed cases for each reviewing radiologist

**Reviewing physician**	**Total assigned cases**	**Total reviewed cases (%)**	**Total not reviewed (%)**
1	254	254 (100)	0 (0)
2	85	71 (84)	14 (16)
3	133	133 (100)	0 (0)
4	388	315 (81)	73 (19)
5	39	23 (59)	16 (41)
6	529	479 (91)	50 (9)
7	261	220 (84)	41 (16)
8	87	56 (64)	31 (36)
9	177	79 (45)	98 (55)
10	288	75 (26)	213 (74)
Total	2,241	1,705 (76)	535 (24)

Numbers in brackets are percentages of total cases for each reviewing radiologist.

**Table 2:** Assigned and reviewed cases by modality

**Modality**	**Total assigned cases (% of total cases)**	**Total reviewed cases (% of modality)**	**Total not reviewed (% of modality)**
Radiography	770 (34)	611 (79)	159 (21)
Fluoroscopy	57 (3)	51 (89)	6 (11)
Mammography	499 (22)	385 (77)	114 (23)
Nuclear Medicine	247 (11)	222 (90)	25 (10)
Ultrasound	668 (30)	436 (65)	232 (35)
Total	2,241	1,703 (76)	535 (24)

The reviewing radiologist agreed with the prior report (score = 1) in 1,690 cases (99.1%; 95% CI, 98.6 to 99.5%; Table 3). Positive feedback (score = 0) was given in three cases (0.2%; 95% CI, 0.1 to 0.5%). Twelve cases (0.7%; 95% CI, 0.4 to 1.2%) were evaluated as having discrepant opinions in diagnosis (scores of 2, 3, or 4). A score of 2 was assigned in two cases (0.1%; 95% CI, 0.0 to 0.4%), a score of 3 in nine cases (0.5%; 95% CI, 0.3 to 1.0%), and a score of 4 in a single case (0.1%; 95% CI, 0.0 to 0.3%). Of the cases considered to contain discrepant opinions, there were six cases in general radiography (1.0% of general radiography cases; 95% CI, 0.5 to 2.1%), one case in fluoroscopy (2.0%; 95% CI, 0.4 to 10.3%), none in mammography (95% CI, 0.0 to 1.0%), three cases in nuclear medicine (1.4%; 95% CI, 0.5 to 3.9%), and two cases in ultrasonography (0.5%; 95% CI, 0.1 to 1.7%). Clinically significant discrepancies (scores of 3 or 4) were given in four cases in general radiography (0.7% of general radiography cases; 95% CI, 0.3 to 1.7%), one case in fluoroscopy (2.0%; 95% CI, 0.4 to 10.3%), none in mammography (95% CI, 0.0 to 1.0%), three cases in nuclear medicine (1.4%; 95% CI, 0.5 to 3.9%), and two cases in ultrasonography (0.5%; 95% CI, 0.1 to 1.7%). Ten of 12 (83%; 95% CI, 55 to 95%) of the score 3 and 4 cases were false negative findings. These included five in general radiography (calcified pleural plaque, remote thoracic compression fracture, talar osteochondritis dessicans, a stable calcified scapular lesion, and findings indicative of COPD), one in fluoroscopy (radio-opaque gallbladder calculi), three in nuclear medicine (a horseshoe kidney missed on a bone scan, and inferolateral ischemia and dilated left ventricle on MIBI scans), and one in ultrasound (small pancreatic tail cyst). One discrepant finding was a false positive in ultrasound (a renal pyramid described as a renal cyst). An error was identified in a nuclear medicine study (renal scan) where the dictated history was incorrect but did not adversely affect image interpretation.

**Table 3:** Scoring of reviewed cases

**Modality**	**Score 0**	**Score 1**	**Score 2**	**Score 3**	**Score 4**	**Total**	**Non-discrepant (0–1)**	**Concordant (0–2)**	**Discrepant (2–4)**	**Clinically Significant Discrepancy (3–4)**
Radiography (%)	0 (0)	605 (99.0)	2 (0.3)	4 (0.7)	0 (0)	611	605 (99.0)	607 (99.3)	6 (1.0)	4 (0.7)
Fluoroscopy (%)	0 (0)	50 (98.0)	0 (0)	0 (0)	1 (2.0)	51	50 (98.0)	50 (98.0)	1 (2.0)	1 (2.0)
Mammography (%)	0 (0)	385 (100.0)	0 (0)	0 (0)	0 (0)	385	385 (100.0)	385 (100.0)	0 (0)	0 (0)
Nuclear Medicine (%)	1 (0.4)	218 (98.2)	0 (0)	3 (1.4)	0 (0)	222	219 (98.6)	219 (98.6)	3 (1.4)	3 (1.4)
Ultrasound (%)	2 (0.4)	432 (99.1)	0 (0)	2 (0.5)	0 (0)	436	434 (99.5)	434 (99.5)	2 (0.5)	2 (0.5)
Total (%)	3 (0.2)	1690 (99.1)	2 (0.1)	9 (0.5)	1 (0.1)	1705	1693 (99.3)	(1695 (99.4)	12 (0.7)	10 (0.6)

Numbers in brackets are percentages of each modality assigned the corresponding score. Score definitions are: 0 = positive feedback; 1 = agreement with original report. 2 = error in diagnosis- not usually made; 3 = error in diagnosis – should usually be made; 4 = error in diagnosis – should almost always be made.

An anonymous survey was completed by all 57 interpreting radiologists. Eighty-eight percent considered the peer review process to be worthwhile. Scores received in reviews were considered appropriate by 79%, and 65% considered the feedback to be appropriate. Reviews prompted 26% to review literature or attend relevant continuing medical education activities. The online cases with clinically significant discrepancy (all grade 3 and 4 cases) were found to be valuable by 67% and of no value by 5%, with 28% not having accessed them.

## Discussion

The scoring system used in our pilot program was the modified RADPEER system[3,4]. Discrepant reviews (score 2, 3, or 4) were provided in 0.7% (95% CI, 0.4 to 1.2%) of total cases. This was somewhat lower than rates reported in the literature by studies using RADPEER, in which the frequency of scores of 2 to 4 range from 2.9% to 4.4% (Table 4). This difference was primarily due to a lower rate of score 2 in our study. The frequency of scores of 2 have been reported by RADPEER to generally correlate with rates of scores 3 and 4 within a radiology practice (r = 0.83), but our results do not fit that profile [4]. We found a score 2 rate of only 0.1% whereas rates in the literature ranged from 1.4% to 3.6%. There are several possible explanations for this based on analyses presented in the literature. One possibility is simply individual tendencies to assign certain scores. Borgestede *et al.* gave examples of score 2 rates for individual radiologists ranging from 0% to 7.3% within the same institution [4]. Another explanation may be the mix of imaging modalities in our study compared to published reports. Score 2 occurs less commonly in mammography, ultrasound, and plain film imaging, which comprised a large proportion of our study mix, than in CT and MRI, which were not included in our pilot [4]. Another reason why our study had fewer scores of 2 may relate to the size of our practice, as it has been reported that the rate of interpretation disagreement declines by 0.5% for each additional 10 interpreting radiologists [4]. Another explanatory variable may be that our study took place in a community setting, where score 2 ratings are reported to average 1.5% less than in academic institutions [4]. A final explanation may be a predilection for our radiologists to give certain cases a score of 1 despite minor disagreement that would have justified scores of 2. This is illustrated by one study that examined written comments for score 1 cases and found that some should have been given scores of 2 based on feedback that included minor criticisms of the prior report, indicating there was a tendency to under-score less serious interpretive disagreements [19]. As a result of this tendency, some investigators have chosen to group scores of 1 and 2 as concordant, meaning there is no disagreement of clinical significance [23]. When our concordance rate is determined this way, we had 99.4% of reviews in this category, similar to the literature rates ranging from 98.5% to 99.8%. This result is again consistent with a tendency of our reviewers to rate some score 2 cases as score 1.

**Table 4:** Comparison of scoring in current study to published data

**Author**	**O’Keeffe**	Siegle*	**Borgstede**	Soffa**	**Jackson**	**Swanson**	**Bender**
Reference	Current	27	4	5	3	19	22
Year	2013	1998	2004	2004	2009	2012	2012
Grades							
0	0.2	NA	NA	NA	NA	NA	NA
1	99.1	95.6	96.3	96.5	97.1	96.2	96.5
2	0.1	1.4	2.9	NA	2.5	3.6	NA
3	0.5	NA	NA	NA	0.3	0.2	NA
4	0.1	NA	NA	NA	0.1	0.0	NA
Non-discrepant (0–1)	99.3	95.6	96.3	96.5	97.1	96.2	96.5
Concordant (0–2)	99.4	97.0	99.2	NA	99.6	99.8	NA
Discrepant (2–4)	0.7	4.4	3.7	3.5	2.9	3.8	3.5
Clinically Significant Discrepancy(3–4)	0.6	3.0	0.8	N/A	0.4	0.2	NA

*NA* not available.

Values are percentage of cases in each scoring category.

*Siegle *et al.* used a slightly different scoring system, but it has been accepted by RADPEER; values in the table for this paper are as reported in Borgstede [4].

**Soffa *et al.* used a 4-point rating system with nominally different definitions of each score, but they are very close to the RADPEER system [4,5].

In our study, scores indicating errors that should usually or always be made (scores of 3 or 4), considered clinically significant discrepancies, were given in 0.6% (95% CI, 0.3 to 1.1%) of cases. This is consistent with reported values in the literature, which range from 0.2% to 3.0%. Just as with scores of 2, rates in this category have been reported to vary by individual and by institution, to be influenced by the facility type (higher in academic settings compared to community settings), and to vary by imaging modality [4]. The numbers in our pilot study are too low to statistically differentiate clinically significant feedback rates between modalities, but it is worth noting that no negative feedback was given in any mammography case. Similar low discrepancy results in mammography were described in the ACR RADPEER pilot (0.1% scored 3 or 4) and other studies [4,10,24]. This may reflect the standardized procedures used in assessing and reporting mammograms or the fact that the majority of mammographic studies are screening examinations with no significant abnormal findings [10,24]. Rates of clinically significant discrepancies in our study were similar to results in the literature for ultrasound (0.5%), general radiography (0.7%), and nuclear medicine (1.4%) [4]. Our highest rate was in fluoroscopy at 2.0% (95% CI, 0.4 to 10.3%); this is the first time RADPEER review rating have been described for this modality.

A criticism of the RADPEER program and of similar peer review processes has been that the rates of negative feedback, particularly of clinically significant discrepancies, appears lower than might be expected [3-5,13,25-28]. This has been attributed by some to the reluctance of radiologists to criticize colleagues [4]. The negative feedback rates contrast sharply with the much higher levels of disagreement reported in blinded clinical studies comparing inter-observer performance in radiological diagnosis, where disagreement rates up to 30% are more are described [3,5,11,25-28]. In part, this may reflect the fact that the prevalence of abnormal findings is higher in directed research studies while many clinical radiographs are normal. Since most disagreements relate to false negative findings by the interpreting radiologist as judged by the reviewing radiologist, the rate of disagreement will be proportional to the frequency of abnormal findings [27]. In part, the higher disagreements in research studies may also arise from the very nature of the research protocols, where a wider range of findings are systematically evaluated and recorded in contrast to a lesser range in a typical clinical report. There is currently no objective benchmark for an acceptable level of disagreement in clinical practice [5,7,8,14], Given the fact that a number of unexplained variables have been identified to be associated with rates of disagreement, as noted above, there is reluctance to even attempt to define appropriate rates until further information is available [4,13,19,22]. One suggestion to improve the utility of the RADPEER system has been to allow comments in score 1 cases. Given the tendency of radiologists to classify some cases as score 1 when they should have been score 2, the use of comments allows critical input to the interpreting radiologist despite the under-scoring of disagreement [19].

Even though “error” is the terminology used in RADPEER, it is important to note that discrepant reviews simply indicate disagreement between the reviewing radiologist and the initial interpreting radiologist, and does not in itself mean that the interpreting radiologist made a mistake [4]. This points to the potential value of third-party adjudicators to provide consensus, accomplished in our program by review of all score 3 or 4 cases by the QA committee. In our pilot, all of these cases were ultimately considered to be errors. Single adjudicators are probably not sufficient to serve as the gold standard. An evaluation of 25 clinically significant discrepant cases (scores of 3 or 4) found that inter-observer agreement by multiple subspecialty reviewers was only slight to fair (kappa values of 0.11 to 0.20) [22]. Consensus evaluation by a multi-person QA committee will likely produce a more acceptable adjudication [18].

An aspect of the modified RADPEER system is the addition of score 0 to the original regime to allow positive feedback [3,4]. Such feedback can potentially play a role in improving quality by reinforcing performance regarded by a colleague as exemplary. In our study, only three cases (0.2%) received positive feedback. While this is a low proportion, it should be noted that this was greater than the number of cases that received a score of 4 (indicating a missed diagnosis that should almost always be made). In the future, as radiologists become more familiar with routine peer review, positive feedback may become more common.

In addition to concerns about the low rate of discrepant scoring, another criticism of radiology peer review is the low completion rate by reviewers in RADPEER and other programs. In the RADPEER pilot, less than 10% of participating radiologists completed more than 200 cases despite the fact that participation was voluntary [4]. By way of explanation, it has been stated that radiologists resist time and resource commitments for additional activities outside the normal work activities, even small ones, due to work burden and costs of implementing such programs [4,11]. In our study, we encountered similar issues with completion of reviews, even though our program was designed to be as minimally intrusive and time-consuming as possible, and despite the fact that participation was voluntary, which might be expected to enlist the most enthusiastic partners. Our median non-completion rate was 18%, with a range from 0% to 74%. Twenty-four % (95% CI, 22 to 26%) of assigned peer reviews were not completed and four of ten radiologists completed less than two-thirds of assigned cases. A recent report of another workstation-based peer review program found an overall one-year average of 53%, much lower than our 76% compliance rate [19]. In our study, the reasons for non-completion were not formally recorded during the pilot period, but some systemic issues were apparent, and may have similarly affected RADPEER or other peer review programs, underscoring the value of undertaking a pilot project for troubleshooting purposes prior to instituting a full peer review program. Some cases selected for random review could not be evaluated as the cases were chosen from the RIS and many of them did not have corresponding images in the PACS, while other cases did not have an accessible prior report in the RIS. The RIS and PACS were implemented two to three years prior to the pilot study so that prior cases predating implementation of these programs were not accessible. Early in the project, procedures that were non-evaluable were presented for review, such as therapeutic joint injections, and these types of procedures were eliminated from subsequent computer selection. In some circumstances, such as gastrointestinal fluoroscopy, the case selected was not a relevant prior as the computer program did not distinguish between upper GI and lower GI examinations. Another contributor to non-completion may have been the option to delay a review. Of the reviewed cases, 94% (95% CI, 93 to 95%) were completed within 15 minutes of assignment, indicating that once a radiologist had committed to a review, it was done during the reporting of the current case. If a review was not done immediately upon presentation, however, the reviewing radiologist was reminded when the current dictation was completed. If the review was delayed further, the case was saved pending completion of the review. The radiologist was then required to sign into the peer review program and manually search for the case in PACS, a less efficient option. The program is being modified so that a second reminder will be issued at the time a radiologist verifies a current report, and so that the reasons for non-completion will be documented. All voluntary peer review programs will suffer from similar variability in commitment, although rates will certainly increase if peer review is made a mandatory part of practice or hospital protocol, or if required for certification or other regulatory reasons. One suggestion that has been made is to institute financial incentives or penalties [19]. Another suggestion for review systems integrated into the workstations has been to block the ability to continue with the daily workload until quality assurance reviews are completed [19]. These approaches have not received acceptance as they place a burden on participating radiologists [14,19]. One mechanism shown to improve compliance, and that will likely be more acceptable to the profession, is monthly compliance reports to individual reviewing radiologists. One study found with this approach that compliance rates rose from 42% to 76% over one year [19].

Many radiologists function in environments with little opportunity to systematically identify errors and thus correct knowledge gaps [2,11], but it has been difficult to achieve such opportunities in routine practice [3,4,11,20]. We have described the results of a pilot peer review program which demonstrates an approach that can be applied in clinical practice, even in a large radiology group with busy outpatient clinics. A key feature of the pilot program was a review process that was workstation-based and integrated into reporting software so that it was seamlessly incorporated into the normal workday [13,29]. In addition, the procedure was practice-integrated in the manner of the RADPEER program in that the images chosen for review were prior studies related to cases currently being reported, and the review was performed during the regular reporting process [3,4,13]. Since relevant prior reports and prior images are routinely evaluated when a current study is being reported, linking the review process to active cases reduces the time and work burden [3,4,13]. Through computerized automation, no work was required in pulling and collating cases, and the cases were randomly selected and representative of the practice [3,7,8,13,18]. The review itself was performed within the reporting software so that there was no need to record paper or online review forms [4,29]. Assessment of data using our approach was done locally, so that interpreting radiologists could be immediately informed of their peer results, individual and aggregate case data could be prepared on an as-needed basis, and cases could be easily identified for discrepancy rounds, case-sharing, and other educational initiatives 11, 13, 19, 20, 21.

In addition to concerns about low discrepancy rates and low review completion rates, a number of other critiques have been leveled at the use of peer review in radiology and some of these remain as limitations of our protocol. One is that this approach does not delve into the ultimate clinical diagnosis so that it can be correlated with radiological findings to provide definitive feedback. The response to this is virtually universal among those involved in quality assessment in radiology: there are no accepted definitions of what constitutes the gold standard for evaluation of most imaging findings and it would be excessively time-consuming and cost-prohibitive to attempt to track cases [1,2,4,8,11,12]. Such an accuracy-assessment program will probably only occur if mandated and funded by external sources. A related criticism is that most peer review systems do not incorporate an evaluation of the clinical significance of radiological discrepancies. For example, among our cases scored 3 or 4 was a stable calcified scapular lesion and inferolateral ventricular ischemia, which would clearly differ in clinical importance. The most recent iteration of RADPEER includes the option for rating clinical significance, but there has been poor uptake [3,4]. The reason is straightforward: it is often difficult to judge the clinical significance of a particular radiological finding in the absence of full clinical information, which is rarely available at the time of reporting. Given the experience of RADPEER, we chose to not include rating of clinical significance in our peer review process.

Our study did not include either CT or MRI studies simply because our pilot program was done in outpatient clinics, and in our health region these modalities are primarily hospital-based. Many published studies have included these modalities, so there is no reason that they should not be included in peer review programs [4,5,19,23,27,30]. By virtue of their increased complexity and the fact that they are often secondary tests done to follow-up an abnormal result or suspected abnormal result on other imaging, a higher disagreement rate is to be expected, and this is what has been found [4,5,19,23,27,30]. We believe that all imaging modalities, including those that are more operator-dependent, such as fluoroscopy and ultrasound, can be incorporated in peer review, although special attention may be needed in choosing and evaluating these cases [4,5,23].

Two-thirds of our interpreting radiologists reported that the review feedback they received was appropriate, 26% reported that the reviews prompted them to review literature or attend educational events, and 67% found the online presentation of score 3 and 4 cases to be valuable. Based on rates of clinically significant discrepancies of 0.2 to 0.8% (Table 4), it requires 125 to 200 peer reviews to find one important disagreement. Is this disruption, with the associated time commitment, expense, and potential exposure to regulatory and legal repercussions, worth the perceived educational benefit? Studies are needed in two areas to better define the appropriate role for peer review in radiology. First, the financial costs of peer review need to be quantified, as they are borne by radiologists or academic departments. Second, the outcomes of the peer review process need to be determined to see whether future practice performance is improved. With such data, cost-effectiveness of peer review can be determined. For now, the approach of the American College of Radiology and of many other groups is that “peer review has become an essential component of a comprehensive radiology department quality assurance program” [19]. Implementing a workstation-based computerized program we have described is one way to effectively incorporate peer review in an active clinical practice.

## Conclusions

Peer review should identify opportunities for quality improvement, facilitate improved outcomes, and contribute to increased competence [7,8,13,19]. Review of possible errors made by colleagues is a recognized learning opportunity for the reviewing physician, the interpreting physician, and those participating in discrepancy rounds or related educational activities [18]. Our pilot project has demonstrated one way in which this can be accomplished using a workstation-integrated computerized system that randomly selects prior cases for review based on cases currently being reported. This approach minimizes time and work impact by blending reviews into the normal workday. Cases were drawn fairly equally from different imaging modalities and the selection process is intrinsically random, avoiding bias. Discrepancy rounds or virtual discrepancy rounds that present cases with scores of 3 or 4 facilitate dissemination of information, with the majority of our radiologists feeling such rounds were valuable. Our radiology group has now instituted workstation-integrated peer review with mandatory participation for all radiologists in the clinic-based part of the practice and is actively working towards establishing a similar review system in our hospital departments including all modalities. Peer review should be considered by all radiologists as a means to reduce errors and improve consistency. If widely adopted, this could demonstrate to the public and governments that the radiology profession is committed to the highest standards of clinical care. The cost-effectiveness of peer review needs further study, but for now remains the primary quality assessment tool in radiology.

## Abbreviations

ACR: American College of Radiology; CI: Confidence interval; MIC: Medical Imaging Consultants; PACS: Picture archiving and communication system; QA: Quality assurance; RIS: Radiology information system.

## Competing interests

Todd Davis is an employee of Intelerad. The other authors have no competing interests.

## Authors’ contributions

MMO participated in study design, data analysis, and data interpretation, and contributed to drafting the manuscript. TMD participated in study design, collected raw data, and provided critical revision of the manuscript. KS participated in study design, data analysis, and data interpretation, and contributed to drafting the manuscript. All authors have read and approved the manuscript.

## Authors’ information

MMO and KS are affiliated with the Department of Radiology and Diagnostic Imaging, University of Alberta, and with Medical Imaging Consultants, Edmonton, Alberta, Canada. TMD is an employee of Intelerad, Montreal, Quebec, Canada, and developed the Intelerad Peer Review software.

## Pre-publication history

The pre-publication history for this paper can be accessed here:

http://www.biomedcentral.com/1471-2342/13/19/prepub
